# An AI Perspective on Counseling Supervision

**DOI:** 10.3390/bs16061038

**Published:** 2026-06-22

**Authors:** Emily A. Brinck, James L. Soldner, Hung Jen Kuo, Scott A. Sabella, Trenton J. Landon, Charles P. Bernacchio, Elizabeth A. Boland

**Affiliations:** 1 Wisconsin Center for Education Research, University of Wisconsin-Madison, Madison, WI 53706, USA; 2 School for Global Inclusion and Social Development, University of Massachusetts Boston, Boston, MA 02125, USA; james.soldner@umb.edu; 3 Department of Counseling, Educational Psychology, and Special Education, Michigan State University, East Lansing, MI 48824, USA; kuohungj@msu.edu; 4 Department of Counseling, School, and Educational Psychology, State University of New York at Buffalo, Buffalo, NY 14260, USA; sasabell@buffalo.edu; 5 Department of Special Education and Rehabilitation Counseling, Utah State University, 2865 Old Main Hill, Logan, UT 84322, USA; trent.landon@usu.edu; 6 Department of Counselor Education, University of Southern Maine, Portland, ME 04104, USA; charlieb@maine.edu; 7 Department of Health and Community Studies, Western Washington University, Everett, WA 98201, USA; bolande@wwu.edu

**Keywords:** supervision, artificial intelligence, ethical and legal issues, supervisor, supervisee

## Abstract

The increased use of technology-assisted distance counseling practices is one result of COVID’s impact on behavioral health, including in counselor education and the delivery of supervision. First, technology-assisted distance supervision needed for “real time” communication grew. Furthermore, there is an emergence of artificial intelligence (AI) technologies that have the potential to contribute to aspects of supervision; however, current evidence remains emerging, context-dependent, and at times mixed, warranting cautious interpretation of their effectiveness. The article offers an overview of using AI in clinical supervision, examines the benefits and potential concerns of AI from different perspectives, and considers the significance of using AI in counseling supervision. The role of AI is discussed as applied to counseling supervision including the use of AI tools, such as chatbots and reasoning AI, to detect and track sessions, note behavioral and emotional cues, aid/monitor communication and feedback, while also attending to ethical and legal consideration for its use. The article will report a range of benefits for supervisors and trainees using AI—for example, by enhancing data-driven supervision decisions, analyzing feedback trends, providing more efficient administrative monitoring, flexible/remote support, skill development, and promoting ethical decisions and self-reflection. Special attention is given to the challenges of using AI in supervision, including risks of undervaluing intuition and qualitative insights, potential for algorithms to reinforce systemic biases, risks of replacing human interaction, as well as non-compliance with HIPAA, FERPA, and ethical guidelines in data storage and privacy. The article will discuss privacy concerns, depersonalized feedback, and increased judgment-driven anxiety despite needed empathy when using AI as a tool for clinical supervision. Recommendations will also be offered for effective, ethical integration of AI in counseling supervision.

## 1. Introduction

The interest and the use of artificial intelligence (AI) in behavioral health grew considerably as technology’s important role emerged during the COVID pandemic. Resulting from this and other factors, there has been a call for increased research on comprehensive frameworks for AI chatbot integration, technology-assisted distance supervision (TADS) models for counselors in-training, and further exploration of AI’s potential in the counseling profession (Maurya & DeDiego, 2023). There is a need in the counseling discipline to critically examine the ethical and beneficial integration of AI in counselor education, focusing on training programs and guidelines to ensure responsible and effective integration, particularly in clinical supervision. There are other researchers who value supervision for preventing burnout and ensuring the effectiveness of new counseling trainees’ interventions, asserting that AI may enhance aspects of supervision; however, empirical findings to date are limited and sometimes inconsistent, indicating that AI should be viewed as a developing supplement rather than a proven enhancement. Furthermore, AI technologies may offer more possibilities for developing clinical practices, with supervision being particularly suited for this type of automation (Cioffi et al., 2025).

A review of studies on clinical supervision in the counseling specialty area of rehabilitation counseling shows ethical practice has been researched often, yet using AI as an innovative technology for TADS and its application with different supervision modalities being considered here addresses two areas that are infrequently studied in clinical supervision based upon this comprehensive review (McCarthy et al., 2025). From a theoretical framework, special consideration should be given to adapting AI’s application through a developmental model theory of supervision, e.g., using the Integrated Developmental Model (IDM) by Stoltenberg and Delworth (Maki & Delworth, 1995). This theory is structured on levels of awareness (counselor and client perspectives)—motivation that centers on relationships, for example, learning different perspectives in a working alliance; being competent, for example, using technology to support clinical practice; and finally, autonomy towards increasing self-confidence and using supervision to set the agenda that meets all parties’ needs. Increasing awareness in a therapeutic relationship could be influenced through AI-enhanced feedback that could be more effective in addressing resistance which requires supervision guidance (Borders & Brown, 2022). The insight that supervisors learn to extract from AI could be used to determine the motivation the clients and counselors have, as well as supervisors, to strengthen the working alliance. As supervisors use AI to provide evidence of competency, they can support a supervisee’s autonomy to affirm an agenda in sessions for counseling, as well as for supervision. It is possible strategies would help within certain supervision modalities, e.g., individual, dyadic, group or peer, to interject with AI in ways that strengthen facilitation of the supervision process. The supervisor’s use of AI would need to assure that the parties can increase awareness of each other while seeing a motivation in growth by understanding each other’s perspectives. Utilizing different modalities, supervision must apply AI in ways that will reinforce greater autonomy as well as leadership that is recurring (McCarthy, 2013; Schultz et al., 2002). We consider the benefits that using AI can offer, given the development of supervisees and supervisors while also realizing its challenges and considerations, which will be discussed later in the article.

Of note, the search methods for journal articles included in the current article involved a broad review, though the literature review process had a limited scope in the writing of this conceptual manuscript. The search had to be broader given this subject is new to the field and so we used the term “AI in human services” searching various databases including APA PsychINFO, CINAHL, ERIC, Academic Search and Cochrane. All articles that pertained to using AI in behavioral health and health-related practice were included given the dearth of literature on this supervision topic.

Furthermore, this paper postulates the role AI could play in clinical supervision of counselors-in-training as well as those prepared to practice. It provides consideration for the benefits of using AI in supervision from different perspectives, as well as the parameters of using AI and the challenges it presents for clinical supervision, and finally, the recommendations for the ethical and effective use of AI in counseling supervision and practice. The following discussion of the role, benefits, and challenges of AI in counselor supervision includes commentary on the supervisor working alliance (SWA), which has been recognized as a pivotal common factor cited across more than a half-century of supervision theory, practice, and research (Watkins, 2014). The SWA may be contextualized as a composite of bond, collaborative goal orientation, and tasks (Bordin, 1983) that combines relational dynamics with supervision process activities to enhance counselor learning and client care (Watkins, 2014). This intersection of human elements with operational functions may be at the crux of debate over AI in counselor supervision as there is the promise of technology increasing efficiency and effectiveness, though with a potential loss of humanness.

Recently, the National Board for Certified Counselors (NBCC) issued core principles on ethical counseling practice and client well-being in the use of AI, emphasizing that while AI can expand opportunities and enhance counseling practice, it also presents ethical challenges for the counseling discipline (NBCC, 2024). These principles are the clinical tenets outlined by NBCC that align with and reinforce directives from its code of ethics. Within the clinical tenets, NBCC specifies directives for supervision and consultation related to AI use. These include: (a) maintaining knowledge of AI tools utilized by supervisees, (b) articulating whether and in what manner AI will be integrated into supervision, (c) consulting with experts when unfamiliar with the technical details of an AI system and engaging supervisees in dialog about its use, and (d) documenting all consultations concerning AI tools within supervision records.

## 2. The Role of AI in Counseling Supervision

The development and widespread use of AI technologies, including AI applications and popular chatbots such as ChatGPT, have exploded over the past several years. A chatbot is considered a computer program that uses language processing and machine learning to decipher natural language based on user input and respond in a conversational and understandable manner (Maurya & DeDiego, 2023). An important feature of chatbots is their contextual understanding of conversation and ability to directly engage with users in natural, contextually relevant dialog and across varied domains. An AI application can be considered any program or software that uses AI to conduct tasks that customarily require “human intelligence” including decision-making, problem solving, and learning, in general. AI applications perform a wide range of tasks such as data analysis as well as automated and natural language processing. In addition to chatbots as one of the most popular and widely used AI applications, other examples of commonly used AI applications include virtual assistants (e.g., Google Assistant, Siri, Alexa), image and speech recognition (used to identify users’ faces, objects and transcribe spoken language), product recommendations (suggests to user products on platforms such as Amazon or social media sites such as Instagram or Facebook), and personalized content (i.e., social media and entertainment and/or educational platforms such as YouTube and Google), to name a few. Considering their significant flexibility and usage, AI applications have become increasingly popular and are being used both personally and professionally and in a wide variety of fields including supervision and broader counseling and clinical settings (Norton, 2025; Park et al., 2025).

There are a range of AI tools currently being used in counselor education and supervision. One of the most widely used AI tools includes chatbots, such as ChatGPT, Google Gemini, Microsoft Copilot, and Meta AI. For example, ChatGPT and other chatbots can be used to create case vignettes by generating context-specific prompts. ChatGPT can also be used for assessment purposes with the development of test items and other forms of assessment based on the nature of the course and subject area. Another area that ChatGPT has been used for is practicing counseling skills via simulated role plays, widely considered a common training technique used in counselor education and supervision (Norton, 2025; Park et al., 2025). In essence, ChatGPT can play the role of a simulated client for the purposes of practicing counseling skills in a safe learning environment. AI applications have also recently been used for counseling supervision and practice documentation purposes (Peavy et al., 2024; Sheperis & Sadeh-Sharvit, 2023). Furthermore, AI applications have been used to produce case notes for the compilation of real time and post-session data as well (Maurya & DeDiego, 2023). This data could include real-time transcription of counseling and/or supervision sessions, including the ability to insert comments and feedback at specific times during the session. These observations and feedback could be provided in real-time or post session and for learning and skill development purposes for supervisee and practicing counselors. At the same time, these tools must be understood within the broader limitations of AI, highlighting that human clinical judgment remains essential for interpreting outputs, contextualizing feedback and ensuring that technology enhances rather than replaces professional discernment. For a more detailed overview of specific, available AI applications currently being used in counselor education and supervision, see Table 1.

There are a range of evolving and important ethical and legal considerations with the integration of AI into counseling and supervision domains (Norton, 2025). In 2024, the NBCC published a document outlining key ethical principles for AI in counseling. This document highlights the responsible and ethical use of AI in upholding core principles of counseling ethics and client well-being, including: (a) accountability, (b) client welfare, (c) competence (AI), (d) competence (Clinical), and (e) confidentiality. In addition, the NBCC outlined both general tenets and clinical tenets that corresponded with directives from the NBCC Code of Ethics. The general tenets included the ethical mandates of transparency and consent, competence and oversight, and accountability. The clinical tenets included directives centered on client welfare, confidentiality, telemental health, supervision and consultation, counselor education, testing and appraisal, research and reporting, and gatekeeping and advocacy. Regarding supervision and consultation specifically, the NBCC listed the following directives: (a) Supervisors should be knowledgeable about AI tools used by supervisees (NBCC directive 41), (b) Supervisors should explain if and how AI will be used in supervision (NBCC directive 42), (c) Address ethical use of AI in supervision agreements (NBCC directive 42), (d) Consult experts if unfamiliar with details of an AI system and discuss with supervisees (NBCC directive 5) and (e) Document consultations about AI tools in supervision notes (NBCC directive 50; NBCC, 2024).

In addition to the clarifications and guidance offered by the NBCC, the American Counseling Association (ACA) recently convened an AI work group relying on research-based and contextual evidence, the ACA Code of Ethics, as well as clinical knowledge and skill to develop 13 distinct recommendations for the use of AI in counseling practice. These recommendations include the following: (1) Learn more about the essentials of AI, its subfields, and its applications to mental health (see C.2.a of the ACA Code of Ethics requiring counselors to practice within their boundaries of competence), (2) Stay open, informed, and educated on best practices in AI, (3) Avoid over-reliance on AI, (4) Recognize that AI may contain bias and be capable of discrimination, (5) Career counselors and those who address employment issues should stay informed about how automation is shaping the world or work, (6) Advocate for transparency in AI algorithms, (7) Maintain transparency and informed consent, (8) Leverage AI for data-driven insights, (9) Ensure data security and privacy, (10) Counselors should empower clients to communicate about their AI use, (11) Supervisors can use AI to enhance the development of supervisees, (12) Counselors must understand the limitations of AI in diagnosis and assessment in all counseling settings, and (13) Consider conducting research on the intersection of AI and counseling (ACA, n.d.-b). For an additional description of recommendations, see the subsequent section devoted to recommendations for ethical and effective AI integration in supervision.

## 3. Benefits of AI in Counseling Supervision

AI has gained significant attention across many clinical sectors, including counseling. While early applications suggest potential utility in supervision, the evidence base is still developing, and findings regarding effectiveness vary depending on context, implementation, and outcome measures. Utilizing AI to augment professional output and workplace efficiency has also gained a surge in popularity across all professions. Clinical supervision in professional counseling is no exception. While the field of counseling has traditionally emphasized the human relationship at the core of supervision, researchers and practitioners are increasingly exploring how AI can function as a supportive tool without replacing the essential human presence. AI-based tools are in their early stages of integration within clinical supervision, primarily in training clinics and select community treatment programs. Reports from university telemental health clinics describe the use of AI for case conceptualization, automated session feedback, and task automation such as assessment and progress notes (Peavy et al., 2024; Sheperis & Sadeh-Sharvit, 2023). In this section, we discuss examples of leveraging AI in clinical supervision from a supervisor standpoint and how they can be beneficial.

In recent studies, three major domains of potential benefits for supervisors in using AI in their supervisory practice that are commonly discussed in the emerging literature are: (1) Efficiency, (2) Quality improvement, and (3) Administrative support. Efficiency gains emerge from the automation of routine paperwork and transcription, reducing the time supervisors spend on documentation and enabling more frequent and focused feedback. AI has shown potential to support quality-related processes, such as identifying patterns in counselor behavior; however, evidence regarding the consistency, accuracy, and clinical relevance of those outputs remains mixed. With the help of AI for administrative support, supervisors can generate fidelity metrics and archived recordings for demonstrating adherence to training and regulatory requirements.

In terms of efficiency, AI has been reported to reduce routine workload in some contexts by automating documentation tasks such as assessments and progress notes (Sheperis & Sadeh-Sharvit, 2023). Automated transcription and searchable session records also facilitate faster review cycles, enabling supervisors to provide more frequent, targeted feedback. Table 1 lists several tools that are available for recording and automated transcription with special attention on its privacy and HIPAA-compliant features.

The ability of AI to deliver objective feedback on counselor skills (Sadeh-Sharvit et al., 2023) provides a means of evaluation that could potentially reduce biases. However, the efficacy and accuracy of these types of evaluations are mixed. In a recent study, Millman (2025) compared evaluations generated by counseling professors and AI and discovered that AI-generated evaluations are consistently higher than those of human raters. Moreover, the participants reported that feedback provided by professors is more helpful in four categories, including clarity, practicality, context relevance, and depth of the feedback. On the other hand, a 2025 study with Gestalt psychotherapy trainees found that pre-trained ChatGPT-4 feedback was rated favorably or better than human supervisors in several dimensions, such as professional orientation and empathic approach (Cioffi et al., 2025). Nonetheless, leveraging AI for evaluation and feedback has produced encouraging results, and with some refinement, the discrepancy between AI and human supervisors can be lessened.

For supporting administrative functions of clinical supervisions, AI is seen mainly as a tool for: (a) scheduling and coordination, and (b) reporting and analytics. AI-integrated systems are used to automate appointment scheduling and reminders for both clients and supervisees. Such intelligent scheduling functionality can optimize resource allocation and coordinate follow-ups, reduce administrative overhead, and minimize missed appointments. According to a recent article (Barbieri et al., 2023), the implementation of an AI-enhanced digital healthcare system could provide patient-focused care and drastically decrease administrative burdens and costs. Similar to behavioral health administration using AI, there would be reduced costs for record-keeping, scheduling appointments, correspondence and other administrative follow-throughs. Besides analyzing individual counseling sessions, AI-based solutions can also be used to analyze and summarize data from multiple sessions and generate performance dashboards. Specifically, supervisors can review AI-flagged portions of sessions, focus feedback, and track progress over time using automated analysis, providing evidence and ensuring compliance with best practices (Millman, 2025).

The following is a case example of how AI could be used as part of the supervision process to consider the feedback that AI provides while using AI to guide the process:

- In this example, Lexi has supervised Jalen for over a year using AI over secure video, encrypted emails and periodic in-person meetings. In their relationship, there have been points of discomfort and disconnection including Jalen getting feedback about a recent session from AI that surprised him. They reviewed taped sessions with a client when Jalen felt overly confident about his technique and response to this client at the time. In a section of case notes provided by AI it became evident in the supervision that certain points raised by AI helped clarify when Jalen silenced the client who was having difficulty being assertive. Then the session transcript revealed his pattern of talking over the client’s feeling of being frustrated and AI could provide alternative responses he could have made to improve the therapeutic relationship. Lexi processed his missed observations, well intentions and reactions with Jalen, which gave them the chance to recognize the value of honest feedback and integrating one’s learning during the use of AI in supervision. This use of AI technology helped foster supervisory alliance and offered opportunities for reflection and professional growth.

Time has been cited as a barrier to providing clinical supervision (Landon et al., 2020). Koçyiğit (2022) also noted a lack of time as a preeminent barrier to providing supervision and identified the length of the supervisory session and the time associated with reviewing session transcripts and audio/video recordings of the supervisee as additional barriers. Koçyiğit noted the potential ethical considerations associated with not attending to these supervisory obligations due to a lack of time. Minimum recommendations for the time needed for effective supervision have been suggested (Ellis et al., 2014) and supervisors are not to pick a supervisory format based purely on convenience to the supervisor (ACES Taskforce, 2011, p. 13). This highlights the need for dedicated time and space to implement clinical supervision appropriately while also recognizing the realities and demands of high caseloads, conflicting schedules, and limited time in a workday. Given that the approach best describing clinical supervision in some counseling practice settings is “as needed” instead of on a recurring and regular basis (McCarthy, 2013; Schultz et al., 2002), the use of AI to enhance the timing and immediacy of skill enhancement seems promising as a way to minimize time demands and provide feedback to counselors almost immediately. This may help capitalize on skill development leading to positive outcomes (McCarthy, 2013) and help alleviate turnover intent in counselors who may otherwise feel unsupported in their position (Sabella et al., 2025). The use of artificial intelligence (AI) to serve as a supplementary resource when immediate supervision is not available may be one way to help bridge potential learning gaps in counselor education and supervision (Maurya, 2023b; Woo et al., 2020).

Objectivity is another critical aspect of clinical supervision (Ladany et al., 2013). Ethical standards of supervision require that supervisors not evaluate or endorse those supervisees with whom they cannot remain objective (ACA, 2014, F.3.d.; CRCC, 2023, I.4.e.). Hutman et al. (2023) found neglectful and callous approaches to supervision to be harmful to the supervisory relationship, and inappropriate feedback, lack of availability, and unresponsiveness from the supervisor were reported by supervisors as inadequate to the needs of supervisees. Any supervisory format that relies heavily on evaluation and the use of negative or critical feedback will adversely impact the supervisory process (Ladany et al., 2013). The use of AI may be a way to decrease the potential of subjective or callous reviews of counselor skill and proficiency. However, the “human” component inherent in the SWA is a critical component of supervisee learning and growth (Ladany et al., 2013).

Another form of technology with the ability to enhance the skill development of counselors and augment traditional methods of instruction is virtual reality (VR; Pope & Hilliard, 2024). The ability of VR to emulate authentic counselor/client interactions and provide opportunities for real time, in vivo experiences has greatly improved and provides more interactive learning than AI alone. Learning enhanced by VR has been shown to increase counselor empathy (Gavarkovs, 2019) and supervisee self-efficacy (Jacobs & McEwen, 2021). Evangelou et al. (2026) examined the combined use of VR and AI to enhance learning outcomes in counselor training. They found that debriefing after simulation-based learning helped increase counselor self-efficacy and competence and argue “… the isolated use of VR is insufficient” (Evangelou et al., 2026, p. 17). Although the use of VR provides opportunities for direction of supervision and immediate feedback (McGhee et al., 2012), supervisors can enhance the learning potential of VR by combining its use with debriefing sessions augmented by AI evaluations of the session to identify areas for learning. Given the lack of experience of early career counselors and students, VR coupled with AI may be a way to help enhance session management and offset feelings of inadequacy and performance anxiety in counseling sessions and improve overall counseling competency and confidence (Evangelou et al., 2026; Jeong et al., 2025).

Although relatively new in its application to behavioral health, the use of generative AI, specifically Chatbots, have shown promise in mental health service provision as they are able to provide a variety of support services, including the reduction in depression and related symptoms (Boucher et al., 2021; Fitzpatrick et al., 2017; Y. C. Lee et al., 2020; Miner et al., 2017; Norton, 2025). The use of AI-generated feedback for supervisees has been shown to help increase counselor-in-training self-efficacy during counseling sessions. The use of AI also enhances ethical principles of beneficence and nonmaleficence as counselors-in-training are interacting with AI rather than real individuals as they develop skills (Jeong et al., 2025). Harm is minimized when mistakes are made and opportunities for learning can be approached from a growth-oriented process. For supervisees who are still learning available resources in a given community, the ability to produce a list of service providers and the services rendered can help create trust and connection with clients. Given the alignment of some apps with specified theoretical frameworks (i.e., Woebot and cognitive behavior therapy), there is the possibility of helping supervisees better implement a theory-driven case conceptualization of clients.

Masamha et al. (2022), in their scoping review of clinical supervision in nursing, found that alternative parallel forms of supervision were perceived as more supportive, more realistic in everyday practice, and more accessible. These forms included peer mentoring, case formulations, multidisciplinary team meetings, and informal sharing of experiences with co-workers to enhance vicarious learning. Such informal supports were described as “more responsive to the clinical realities of everyday work” (p. 2686). This finding is relevant to the integration of AI because it suggests that support for adopting new clinical tools does not need to occur only through formal top-down supervision. Instead, AI competence may also be developed through parallel, practice-based processes in which clinicians discuss AI outputs with colleagues, reflect on case applications in teams, and collectively evaluate the usefulness and limits of these tools in real clinical contexts. In this way, AI integration can be understood not as a replacement for supervision, but as something that may be more safely and effectively incorporated through the same collaborative, accessible, and context-responsive structures that already support clinical learning. Taken together, these findings suggest that while AI may offer meaningful support in specific supervisory tasks, its effectiveness is not yet well-established across settings, and outcomes may depend heavily on how tools are implemented and interpreted within human-led supervision.

## 4. Considerations and Challenges of AI in Supervision

Although AI presents promising applications in supervision, its limitations are noteworthy and require careful, critical examination. These limitations extend beyond technical constraints and include epistemological, relational, ethical, and systemic concerns that may fundamentally shape how supervision is experienced and evaluated (Sheperis & Sadeh-Sharvit, 2023; Rousmaniere, 2014; Rousmaniere et al., 2016).

A key limitation of AI lies in its reliance on pattern recognition rather than true understanding (Haselager et al., 2024; E. E. Lee et al., 2021). AI systems generate outputs based on probabilistic associations in training data, not on lived clinical experience, ethical reasoning, or contextual judgment; as a result, AI-generated feedback may appear authoritative while lacking depth, nuance, or situational appropriateness (Kooli & Al Muftah, 2022). This creates a risk of ‘automation bias,’ in which supervisors or supervisees may over-trust AI outputs despite underlying inaccuracies or incomplete reasoning (Haselager et al., 2024).

Algorithmic bias represents a significant and well-documented limitation (Gupta et al., 2021; Z. Li et al., 2025). AI systems trained on historical or non-representative datasets may reproduce or amplify systemic inequities, particularly related to race, culture, language, and disability (Trigo et al., 2024; Chin et al., 2023). In supervision, this could manifest as misinterpretation of culturally grounded communication styles, inappropriate evaluation of counseling approaches, or skewed performance metrics. These risks are not merely technical but ethical, as they may disproportionately disadvantage already marginalized groups (CRCC, 2023; ACA, n.d.-a).

Beyond technical limitations, AI may alter the relational dynamics central to effective supervision. The supervisory working alliance relies on trust, empathy, and mutual meaning-making elements that cannot be replicated by algorithmic systems (Watkins, 2014). Overreliance on AI-mediated feedback may shift supervision toward a performance-monitoring model, potentially increasing anxiety, reducing openness, and undermining reflective learning processes essential for counselor development (Ladany et al., 2013; Sabella & Friedman, 2024). Al also poses risks in clinical judgment, particularly in high-stakes areas such as risk assessment, diagnosis, and intervention planning. As current evidence suggests variability in AI accuracy, especially in complex or ambiguous cases, reliance on AI-generated recommendations could lead to under- or overestimation of client risk (Elyoseph & Levkovich, 2023). This highlights the necessity of maintaining human oversight as the primary decision-making authority (ACA, 2014; NBCC, 2024).

Practical implantations further limit AI’s effectiveness. These include variability in tool quality, lack of standardization, cost barriers for secure systems, and limited access in under-resourced settings (J. Li, 2023; Moran et al., 2025). Additionally, legal and regulatory frameworks have not kept pace with technological advancement, creating uncertainty around liability, data ownership, and compliance (CRCC, 2026; ACA, n.d.-b).

These conceptual and relational concerns are further reflected in practical, ethical, and legal challenges within supervision. The potential risks of using AI in the supervision process would include possible violations of confidentiality and exposing the privacy of both clients’ personal information as well as that of counselors-in-training. Experts have advised creating policies that will guide the ethical use of AI emphasizing when and how to utilize various AI tools while maintaining the highest standards of ethical conduct in the counseling profession (Maurya & DeDiego, 2023). Considering the requirements of preparation and training of counselors to adhere to specific ethical codes (ACA, 2014) pertaining to supervision and protection of confidential information, new policies will be needed with guidelines for supervisors and trainees, which will result in revising relevant ethical codes in the use of AI technology (Maurya & DeDiego, 2023).

Currently, standard F.2.c in the *ACA Code of Ethics* related to “online” supervision states “When using technology in supervision, counselor supervisors are competent in the use of those technologies.”

When using technology in supervision, counselor supervisors are competent in the use of those technologies. Supervisors take the necessary precautions to protect the confidentiality of all information transmitted through any electronic means.

(ACA, 2014, p. 13)

A similar standard related to technology-assisted distance supervision is included in the CRCC *Code of Professional Ethics for Certified Rehabilitation Counselors* (CRCC, 2023, I.2.c).

A more sophisticated examination of the legal risks using AI would pose to intellectual property and confidential information is providing certain best practices to effectively grasp these risks and effectively manage them (Prinz, 2025). One such best practice will require having a policy that expressly prohibits uploading documents or files containing confidential, proprietary, sensitive, or personal information to generative AI platforms, except to a generative AI platform that is privately operating in a closed, restrictive environment limiting access to only authorized users.

In addition to the *ACA Code of Ethics* (ACA, 2014), the *Best Practices in Clinical Supervision* (ACES Taskforce, 2011) also requires that counseling supervisors engage in sound informed consent practices, as well as provide written documents (in-person or through email), which they must verbally review with their supervisees and that must include a discussion on the use of all technologies, such as AI, at the start of supervision (ACA, 2014, Standard F.4.a; ACES Taskforce, 2011, 1.a.1). In order to effectively sustain the supervisee’s consenting participation, it will require a form of dialog (e.g., Socratic dialog) between supervisee and supervisor that will give the opportunity for both parties to ask questions, begin to develop a working alliance relationship, and discuss any possible concerns that emerge in the use of AI while trusting they can find solutions (Rousmaniere et al., 2016).

According to Maurya and DeDiego (2023), primary considerations for supervisors and training counselors are the secure transmission and storage of clients’ information, informed consent (e.g., obtaining consent of parties before integrating AI in either counseling or supervision sessions), compliance with government regulations regarding client and trainees data protection (e.g., HIPAA, FERPA), data ownership and retention policy (e.g., defining clear ownership of data generated through counseling/supervision interactions, specifying how long the data will be stored and when it will be deleted), and user authentication and authorization (e.g., ensuring that only authorized individuals have access to sensitive or compromising data).

The utilization of AI tools, like ChatGPT, will require verifying AI-generated content for accuracy and completeness that may introduce biases and inaccuracies, which pose greater risks (Maurya & Cavanaugh, 2023). One pilot study used AI when conducting various assessments of clients being served in a university-operated counseling center (Sheperis & Sadeh-Sharvit, 2023). In the supervision process, researchers expressed fear that AI could be used to point out a trainee’s weaknesses or even incorrect practices in the sessions. Supervision must be cautious in trusting the use of AI tools for determining needs for either treatment interventions or assessing safety concerns (e.g., suicide risk) and the possible need for crisis intervention. A study by Elyoseph and Levkovich (2023) compared a human assessment with a ChatGPT hypothetical case vignette assessment and found it consistently rated suicide risk lower than mental health professional ratings across all scenarios and rated mental resilience lower in most conditions, which was higher for actual mental health professionals. Their findings suggest that relying on AI, with its current capabilities, for assessment purposes (e.g., suicide risk) or as a primary decision-making tool in mental health settings may lead to untimely or inadequate responses and even inaction to address such risks.

Conversely, there is considerable evidence suggesting that mental health professionals often over-estimate the suicidal ideation and risk of clients. A systematic literature review by Airey and Iqbal (2022) found that clinicians were overly confident in their ability related to suicide risk assessment. Gale et al. (2016) found that clinicians (particularly doctors and male clinicians) were more likely to assess a higher rate of suicide risk than actually demonstrated. Research has also indicated digital mental health assessment tools and face-to-face mental health services to be equally accurate in the assessment of suicide risk (Nielssen et al., 2022). Supervisors should be aware of the risks associated with being overly confident in assessment of suicide risk on the part of the clinician, and also of an overreliance on a purely technological approach to suicide risk assessment and help supervisees develop this important skill while considering all appropriate tools. These findings provide a contrast to consider when determining the appropriate role for AI when assessing relevant risks among clients.

Research has suggested that the potential for human error exists, resulting in misdiagnosis or inaccurate records when using AI that continues to be a primary ethical concern and consideration (Kooli & Al Muftah, 2022). There is also a likelihood that racial and cultural biases may be perpetuated and even exacerbated with AI (Gupta et al., 2021). Another study involving counselors-in-training reported that about 18% of those who were being supervised voiced skepticism that AI could replace a human therapist, noting concerns for those who are feeling vulnerable, which raises doubts that AI could duplicate a clinician’s ability to provide empathetic care (Boucher et al., 2021 as cited in Fullmer et al., 2023).

A non-peer-reviewed study comparing ChatGPT-4-generated feedback from a trained AI and non-trained AI as well as an experienced clinical supervisor found that the trained ChatGPT-4 earned better ratings on certain aspects of therapy relating to development/support of treatment, technical aspects, and adaptability but was rated lower than the experienced clinical supervisor on emotional and relational dynamics (Cioffi et al., 2025). These findings are preliminary but may have value for guiding supervisors’ use of ChatGPT-4. In another study conducted on the use of an AI chatbot to provide mental health care to counselors-in-training, findings noted the caring connection was not realized by all participants; as evidence, 41% felt AI poorly responded to their emotions and 36% reported issues with understanding user inputs (Fullmer et al., 2023). Overall, the research found the chatbot’s utility was noteworthy in alleviating mental health burdens, for example, anxiety and depressive tendencies through psychoeducation and empathetic support.

Despite how more refined AI is becoming in some areas, such as reasoning or advanced conversational AI being relatively new technologies, only a few studies have been conducted on AI utilization within counselor education and supervision (e.g., Maurya, 2023a, 2023b; Maurya & Cavanaugh, 2023; Maurya & DeDiego, 2023). One suggestion from a pilot study involving the use of AI for telemental health supervision included a need for platforms being used to also provide explanations for AI recommendations as well as requiring parties to be trained to learn AI literacy and technical skills (Sheperis & Sadeh-Sharvit, 2023). There have been similar findings in a study involving training of medical residents, which used simulation-based preparation and discovered a contrast between AI-generated feedback from a simulator and feedback from an actual medical supervisor during a real-life medical practice (Kollerup et al., 2025). Researchers urged for continuous and accurate feedback that resembles interactions between clinical supervisors and residents in “real-world” training scenarios if using AI. Although this was not a counseling supervision study, it still underscores the benefit of the supervisory relationships between both parties that is essential for all health-related disciplines. It was reported that feedback from medical training scenarios using AI is under-explored, yet it is a highly relevant aspect of explainable AI and a significant area in AI utility research for training and supervision of medical residents.

A counselor supervisees’ concerns in the use of AI may involve benefits to work with clients, ethical implications and management of risks, and the use of AI for performance evaluation of supervisees as employees. An initial interest in AI may be how to enhance service delivery and client outcomes, such as increased accessibility of support in remote areas (i.e., transportation and stigma barriers), round-the-clock support, and AI-personalized service delivery according to the individuals’ needs (Ofem et al., 2025). Yet, in practice, administrative responsibilities may be the source of many work pressures (Sabella et al., 2021) and high caseloads, limited time, limited resources, and agency performance demands may lead counselors to seek ways to increase efficiency, or in some cases, to use questionable shortcuts. AI may be helpful when used for information gathering, organization, and synthesis, though using AI as a way of “speeding up-to keep up” (Chubb et al., 2022, p. 1399) with administrative or performance-related concerns may have unintended negative consequences.

As the supervisee works with clients, they are bound by ethical and legal standards related to data storage, privacy, and confidentiality. Neither the ACA (2014), nor the CRCC (2023), have specific ethical standards related to use of AI. Only the American Mental Health Counseling Association Code of Ethics (AMHCA, 2023) presently includes specific standards on the use of technology to support counseling and communication through “the use of artificial intelligence, machine learning, deep learning, or any other human simulation modality” (I.B.6.1). The ACA provides guidance for practicing counselors and recommends that they “clearly inform clients about the use of AI in the counseling process” and “obtain explicit informed consent from clients for the use of AI-assisted tools” (ACA, n.d.-b, para. 10). The CRCC has recently released guidelines on the use of AI in rehabilitation counseling settings as well (CRCC, 2026). It should be noted that these recommended guidelines by the ACA and CRCC are not yet enforceable standards of the respective organizations’ code of ethics. Due to the speed of AI advancement, clear guidelines and laws related to the use of AI-assisted tools with client/student/supervisee data are lagging, including protected or personally identifiable information. Counselors, supervisors, and clients may have a lack of clarity about data security and access through AI-assisted tools and the potential for privacy breaches or use of data by other entities or beyond what is intended. Counselors may be tempted to take advantage of AI’s potential for case management as in client data management, case summarization, or case conceptualization, as reviews of clinical records are time consuming, particularly with high caseloads. Yet, open AI systems have well-discussed data security concerns (J. Li, 2023), and closed systems are expensive and not readily available to practitioners.

In supervision, counselor development is a complex, multilayered process that is influenced by supervisee characteristics, supervisor factors, process dynamics and structure, and the quality of SWA (Bernard & Goodyear, 2021). The SWA is the cornerstone for effective practice and the primary catalyst for growth in supervision (Sabella et al., 2020). The SWA involves a host of human dynamics that may be affected by AI automation, including a risk to undervaluing human intuition and relational dynamics that are keys to learning and integration. This is particularly relevant when counselors are being evaluated, as anxieties are magnified and decisions on “what is said” and “how it is said” may impact whether performance feedback is perceived as debasing or constructive (Sabella et al., 2020). Supervisees may be cautious of an overreliance on performance data sourced from AI that: (a) has unknown accuracy, bias, and methodology; (b) reduces them to a set of data points; or (c) may neglect important humanistic elements such as delivery of feedback in a tactful, affirming, and developmentally appropriate manner. Empathetic listening and responding may mitigate supervisee anxiety and improve openness to feedback (Sabella & Friedman, 2024); these skills may be simulated but are comparative weaknesses of AI models.

A related concern in supervision and counselor evaluation is the recording and use of performance data that is generated or supported by AI. Supervision Best Practices Guidelines state that supervisors should maintain supervision documentation in a way that protects the privacy and confidentiality of the supervisee. These best practices also advise transparency in the supervision process, so that the supervisee clearly understands the evaluation methods, how the information will be used, and the potential consequences (Borders, 2014). If AI is used in performance evaluation or analysis, this should be clearly communicated to the supervisee, including a discussion of the potential for unknown consequences to their privacy, confidentiality of information, or even consequences to employment or future employment if data were released.

Beyond privacy issues, AI is known to generate discriminatory, stereotyping, and misleading content that may be due to faulty algorithmic operations (Z. Li et al., 2025) or through reproducing biases and prejudices that are sourced from historical data (Trigo et al., 2024). Therefore, it is critical that counselors and supervisors remain apprised of the inherent risk in evolving AI technologies and adhere to their ethical responsibilities to minimize potential harm to clients in marginalization, racism, and discrimination (CRCC, 2023). An ACA (n.d.-b) taskforce recommends that counselors consider researching the intersection between AI and counseling so they may increase the understanding of ethical use of AI with diverse populations. Practitioners are expected to assess AI tools before selecting them for use and obtain appropriate informed consent while properly informing clients of the known and potential unknown risks and benefits of using AI tools in service delivery (ACA, n.d.-a, n.d.-b). This guidance seems equally relevant to the supervisor–supervisee process where there is an inherently hierarchical structure and thus the potential for bias influencing the process.

### Recommendations for Ethical and Effective AI Integration in Supervision

As AI tools become increasingly integrated into counseling and clinical supervision, it is essential to establish ethical and practical guidelines to ensure their responsible use (Norton, 2025). While all of the following recommendations contribute to ethical and effective AI integration, several represent foundational requirements such as maintaining human supervisory authority, ensuring transparency and informed consent, and safeguarding cultural responsiveness and equity. Other recommendations, including opportunities for opt-out, collaborative tool development, and ongoing evaluation processes, serve as important enhancements that strengthen implementation once these core elements are in place. This prioritization helps supervisors focus first on the practices most essential to protecting supervisee welfare and maintaining ethical standards.

AI should be viewed as a complement to, rather than a substitute for, the human elements that define effective clinical supervision (Cioffi et al., 2025). While AI can offer valuable support, such as organizing case notes, identifying patterns in supervisee behavior, or suggesting evidence-based interventions, it lacks capacity for empathy, contextual understandings, and ethical discernment that human supervisors bring to the supervisory relationship (Chopik et al., 2017). These relational and reflective dimensions are foundational to the SWA, encouraging professional growth, promoting emotional safety, and enhancing ethical decision-making (Edwards, 2024; Park et al., 2025). AI-based recommendations, without the moderating and guiding effect of the relationship espoused in the SWA, may harm the supervisee’s development as the feedback may be interpreted as overly negative. This deficit-based approach to supervision may lead to negative self-evaluation by the supervisee and an internalized message that they are not progressing.

As such, supervisors must remain the central figures in guiding supervisees, using AI-generated insights as one of many tools to inform their clinical judgment (Gross et al., 2017; Haselager et al., 2024). For example, an AI tool might flag inconsistencies in documentation or suggest relevant literature, but it is the supervisor’s responsibility to interpret these outputs within the broader context of the supervisee’s development, client needs, and cultural considerations. Overreliance on AI risks reducing supervision to a transactional process, undermining the nuanced mentorship that supports counselor identity formation and ethical practice (E. E. Lee et al., 2021; Norton, 2025; Thakkar et al., 2024).

Transparency is a cornerstone of ethical supervision, and its importance is amplified when integrating AI into supervisory practices (Bales & Stone, 2020; Miric et al., 2020). Supervisees must be fully informed about the presence and function of AI tools within the supervisory process. This includes clear, accessible explanations of what data is being collected (e.g., session transcripts, performance metrics), how the data is processed and stored, and who has access to the outputs generated by AI systems (American Psychological Association, n.d.). Importantly, these transparency practices are intended primarily to address issues of data security, privacy protection, and information governance, rather than to introduce additional ethical evaluation beyond existing supervisory standards.

Informed consent should not be treated as a one-time formality but as an ongoing dialog. Supervisors should provide supervisees with opportunities to ask questions, express concerns, and revisit their consent as the use of AI evolves. Consent forms explicitly outline the scope and limitations of AI use, including whether AI-generated feedback will influence evaluations, how long data will be retained, and what safeguards are in place to protect confidentiality (Aydin, 2025). Supervision contracts should also clearly specify how AI will be integrated into the supervisory process, detailing the types of data that will be collected or generated, such as session summaries, performance analytics, or draft feedback, and the purposes for which this information will be used. These contracts should clarify whether AI-generated outputs will contribute to formative or summative evaluation, outline the procedures available for correcting or challenging inaccurate AI-generated content, and define the limits of supervisor oversight when AI tools are involved (Barnett & Molzon, 2014; Falender & Shafranske, 2023). Supervisees should also be informed of their rights regarding data generated about their performance. This includes the right to know what data is being stored, the right to access or review AI-generated records, the right to request corrections to inaccurate or misleading outputs, and the right to opt out of certain forms of AI-based analysis when feasible without compromising program requirements. Clarifying these rights not only strengthens ethical practice but also promotes transparency and trust in supervisory relationships that incorporate AI. Supervisees should be given the option to opt out of AI-supported supervision when feasible, without fear of penalty or diminished learning opportunities. This respects their autonomy and acknowledges the potential discomfort or mistrust some individuals may feel towards emerging technologies (NBCC, 2024; Park et al., 2025).

To ensure that AI tools used in supervision are ethically sound, culturally responsive, and practically relevant, it is essential that counseling professionals are actively involved in their development (Beg, 2025; Chin et al., 2023). Too often, AI systems are created in isolation from the contexts in which they are deployed, leading to tools that may inadvertently reinforce bias, overlook critical nuances in human behavior, or misalign with the values of the counseling profession (Young, 2024). These risks are especially pronounced when AI systems are trained on datasets that underrepresent marginalized groups or encode historical inequities, which can result in skewed performance analytics, misinterpretations of communication styles, or differential error rates across cultural groups. Supervisors therefore have an ethical responsibility to critically evaluate AI tools for potential algorithmic bias and cultural insensitivity before integrating them into supervision. This evaluation should include examining whether the system’s training data reflect diverse cultural and linguistic backgrounds, assessing how the tool performs across different demographic groups, and reviewing whether the AI’s feedback mechanisms privilege certain communication norms or counseling approaches over others. Supervisors should also consider how AI-generated outputs might disadvantage supervisees from marginalized backgrounds, for example, by misreading culturally grounded expressions of emotion, penalizing dialectical or accented speech, or interpreting relational styles through a monocultural lens (Dervin & R’boul, 2025). To mitigate these risks, supervisors can implement safeguards such as cross-checking AI-generated feedback with human judgment, providing supervisees opportunities to contextualize or challenge AI interpretations, and ensuring that AI outputs are never used as the sole basis for evaluation (Dohotaru et al., 2025). By taking these steps, supervisors help ensure that AI applications enhance rather than undermine equity, cultural responsiveness, and fairness within supervisory relationships.

By engaging counselors, supervisors, and counselor educators in the design, testing, and refinement of AI tools, developers can better understand the ethical frameworks, relational dynamics, and cultural considerations that define effective supervision. This collaboration helps ensure that AI systems are not only technically robust but also aligned with core counseling principles such as respect for autonomy, social justice, and multicultural competence (NBCC, 2024).

Importantly, the development of AI tools should be guided by established ethical standards, such as those outlined in the counseling codes of ethics (e.g., ACA, 2014; CRCC, 2023). These codes emphasize the importance of client welfare, informed consent, cultural sensitivity, and the responsible use of technology in counseling practice. Integrating these ethical guidelines into the design process helps ensure that AI tools support, rather than undermine, the supervisory relationship and the broader goals of counselor development (Norton, 2025).

Involving counseling professionals in AI development can help identify and mitigate potential harms early in the design process. For example, professionals can provide insight into how language models might misinterpret clinical language, how data might be misused, or how certain populations might be disproportionately impacted by algorithmic decisions. Their input is critical in shaping algorithms that are transparent, fair, and adaptable to diverse supervisory contexts (Spytska, 2025).

The ethical and effective integration of AI into supervision hinges on the preparedness of both supervisors and supervisees to engage with these tools critically and competently. According to CACREP standards, all supervisors of field experiences, no matter what modalities they use, must have relevant training for in-person and/or distance counseling supervision, and in the technology utilized for supervision in addition to holding pertinent credentials (CACREP, 2024). To help those supervisors become competent, the accredited programs must provide professional development opportunities to fieldwork site supervisors for all program delivery types. The costs will vary for this training, particularly to include AI, depending on whether it is offering courses or a series of workshops. Training is not merely a technical necessity; it is a professional imperative that ensures AI is used in ways that enhance rather than compromise the supervisory process. Training should begin with foundational knowledge about how AI systems function, including their capabilities, limitations, and potential sources of bias (Norton, 2025; Timmons et al., 2023). Supervisors and supervisees must learn to interpret AI-generated outputs with discernment, recognizing that these tools are not infallible and should never replace clinical judgment. For example, if an AI system flags a supervisee’s documentation as inconsistent, the supervisors must evaluate whether the concern is valid within the context of the case, rather than accepting the output at face value (Lewis & Clutterbuck, 2019).

Additionally, training should emphasize ethical considerations, including confidentiality, data security, and the responsible use of technology (Norton, 2025). Supervisors should be equipped to explain how AI tools align with professional ethical standards, such as those outlined in the ACA (2014) and CRCC (2023) codes of ethics. These codes provide guidance on the use of technology in counseling, including the importance of informed consent, cultural competence, and client welfare, all of which are relevant when AI is used in supervision. Practical training should also include hands-on experience with AI tools, allowing users to explore features, ask questions, and develop confidence in their ability to integrate AI into their workflow. Role-playing scenarios, case studies, and collaborative discussions can help supervisors and supervisees to proactively evaluate AI outputs and make ethically sound decisions based on them (Alghowinem et al., 2024; Pahi et al., 2024).

The integration of AI into clinical supervision must be accompanied by structured and ongoing evaluation to ensure its continued relevance, safety, and alignment with professional standards (Wu et al., 2021). AI tools evolve and become more embedded in supervisory practices; hence, institutions and organizations must establish formal mechanisms for periodic assessment and oversight. These evaluations should address multiple dimensions of AI use, including its impact on supervisory relationships, the accuracy and usefulness of its outputs, and its influence on decision-making processes. Regular reviews can help identify whether AI tools are enhancing supervision or introducing unintended consequences, such as over reliance, misinterpretation, or inequitable outcomes (Sheperis & Sadeh-Sharvit, 2023). These reviews should be conducted by interdisciplinary teams that include supervisors, supervisees, technologists, and administrators to ensure a balanced and informed perspective (Moran et al., 2025).

User feedback should be a central component of oversight. Supervisors and supervisees who interact with AI tools on a regular basis are uniquely positioned to identify strengths, limitations, and areas of improvement (Moran et al., 2025; Park et al., 2025). Institutions should create accessible channels for collecting this feedback and incorporate it into the refinement of AI systems. This iterative process helps ensure that AI remains responsive to the needs of its users and adaptable to changing supervisory contexts (Haque & Rubya, 2022; Kim et al., 2018; E. E. Lee et al., 2021). Oversight should include monitoring data security and privacy practices. As AI tools often rely on sensitive information, institutions must regularly audit what is stored, accessed, and used. This includes verifying data-handling procedures to comply with applicable legal and organizational standards that ensure safeguards are in place to prevent unauthorized access or misuse (Moran et al., 2025; Park et al., 2025).

## 5. Future Directions

As AI continues to evolve within counseling supervision, a focused and coordinated research agenda is essential to guide ethical, effective, and culturally responsive integration. Several key knowledge gaps warrant systematic investigation. First, empirical studies are needed to compare AI-augmented supervision with traditional supervision models, particularly regarding supervisee development, skill acquisition, and professional identity formation. Longitudinal research should also examine the long-term effects of AI integration on the supervisory working alliance, including whether AI enhances, disrupts, or reshapes relational processes over time.

Building on emerging findings from recent content-analysis work, future research should explore how AI functions across diverse client populations, counseling modalities, technological platforms, and multicultural contexts. This includes examining how algorithmic biases may manifest in supervision processes and identifying strategies to mitigate these risks, especially when working with culturally diverse supervisees and clients. Additional inquiry is also needed to understand whether AI-supported supervision influences client rehabilitation outcomes, and whether certain modalities or technologies benefit more from AI augmentation than others.

Methodologically, the field would benefit from mixed methods approaches that capture both quantitative indicators of supervision quality and qualitative experiences of supervisors and supervisees. Content-analysis methodologies may be particularly valuable for mapping trends in AI-supported supervision and identifying priority areas of empirical study. Although outcome measures for evaluating AI in supervision are still emerging, early work might incorporate social validity indicators or simple evaluative questions (e.g., “Did AI tools enhance the supervision experience?”) as foundational metrics for future, more sophisticated assessment tools. Such efforts will help establish a rigorous evidence base that can guide responsible, effective, and ethically grounded integration of AI into counseling supervision.

## 6. Conclusions

AI is increasingly shaping the landscape of counseling supervision, offering opportunities to support varied aspects of practice, provide objective feedback, and support administrative tasks for all involved. However, its integration must be approached with caution and intentionality. While AI can streamline documentation, assist in case conceptualization, and offer timely insights, it cannot replicate the relational depth, empathy, and ethical discernment that define effective clinical supervision. The SWA remains the cornerstone of counselor development, and AI should serve as a complementary tool, not a replacement, for human judgment and connection.

While AI offers potential benefits in supervision, the current evidence base remains limited and mixed, and its integration must be approached with careful consideration of its significant limitations. Yet these advantages are tempered by significant challenges, including risks of over-reliance, ethical and confidentiality concerns, algorithmic bias, and diminished human connection. Supervisors and supervisees must remain vigilant in ensuring that technology aligns with professional values and ethical standards, particularly regarding informed consent, data security, and cultural responsiveness.

Moving forward, the counseling profession must prioritize collaborative development of AI tools, ongoing training for supervisors and supervisees, and structured oversight to monitor impact and mitigate risks. By embedding ethical principles and maintaining transparency, AI can complement, not compromise, the supervisory process. Ultimately, the goal is to leverage technology in ways that uphold the integrity of counseling supervision, encouraging growth, competence, and client welfare in an increasingly digital world.

## Figures and Tables

**Table 1:** AI-powered and HIPAA-compliant tools commonly used for counseling supervision.

Tool	What It Offers	Privacy/HIPAA & Other Features	Considerations
Tava Scribe (2025)	Real-time transcription + session summary (e.g., Subjective, Objective, Assessment, Plan (SOAP) notes). Automatically deletes raw transcripts after summary.	HIPAA-compliant; includes consent handling. More info: https://www.mentalyc.com/security (accessed on 15 April 2026)	Works well for virtual sessions; in-person support still under development.
Mentalyc (2026)	AI-powered therapy transcription, structuring session notes, insights (therapeutic alliance metrics, etc.). https://www.mentalyc.com/security (accessed on 15 April 2026)	HIPAA-compliant with secure cloud storage, audit logs etc. More info: https://www.tavahealth.com/therapists/tava-scribe (accessed on 15 April 2026)	May require review for accuracy; costs scale with usage.
Supanote.ai (2026)	Converts therapy sessions into structured notes (SOAP) almost immediately.	HIPAA compliance, signed Business Associate Agreements (BAA), encryption etc. More info: https://www.supanote.ai/hipaa-compliant-ai-therapy-notes (accessed on 15 April 2026)	Subscription model; may have limits depending on plan.
Symmara (2026)	Automated transcription + summaries; claims to reduce documentation time by ~70%.	HIPAA-compliant platform. More info: https://symmara.com/ (accessed on 15 April 2026)	Accuracy matters; supervisor editing recommended.
TranscribeMe (2026)	Hybrid human + AI transcription for healthcare; can offer higher accuracy for nuanced language.	HIPAA compliance, confidentiality procedures etc. More info: https://www.transcribeme.com/hipaa-compliance/ (accessed on 15 April 2026)	Slower if human transcription used; higher cost.

Note: AI tools are evolving rapidly, and their features, limitations, and compliance status can change quickly. Supervisors should verify current capabilities, approved use cases, and applicable legal, privacy, and organizational compliance requirements before relying on these tools in practice.

## Data Availability

No new data were created or analyzed in this study. Data sharing is not applicable to this article.
